# Supplementary material for: Impact of a Treatment Guide on Intravenous Fluids in Minimising the Risk of Hospital-Acquired Hyponatraemia in Denmark
Source: J Clin Med. 2023 Aug 3;12(15):5105. doi: 10.3390/jcm12155105 (PMC10420267; doi:10.3390/jcm12155105)
Supplement: Supplementary file 1 [file jcm-12-05105-s001.zip › jcm-2430547-supplementary.pdf]

Supplementary Materials

Treatment guide

The treatment guide was designed so that it fit into the breast pocket of a doctor's coat and was easy to carry around. The treatment guide contained basic information about IV fluids and hyponatraemia, as outlined in the table below. Intravenous fluids contain water, electrolytes, and glucose, and are used to treat conditions such as hypovolaemia, dehydration, and electrolyte imbalance, as well as for maintaining fluid balance.

Page no.	Main information
1	General information about IV fluid administration and monitoring of P-Na and blood glucose
2	Commonly used IV fluids and their tonicity after injection
3	Treatment of hyponatraemia with severe symptoms
4	Treatment of hypernatraemia
5	Risk groups: children, patients with potentially increased intracranial pressure and patients with liver cirrhosis

Pre-intervention questionnaire

[A cross (X) is used to indicate correct answers. References were not part of the questionnaire presented to the participants]

Background questions

1. What is your gender?

- ☐ Female
- ☐ Male
- ☐ Other

---

2. Which of the following categories describe your age? (check only one)

- ☐ 18 – 24 years
- ☐ 25 – 34 years
- ☐ 35 – 44 years
- ☐ 45 – 54 years
- ☐ 55 – 64 years
- ☐ 65 years or older

---

3. How many patients do you treat with IV fluids in an average week? (check only one)

- ☐ 0 patients
- ☐ 1 - 5 patients
- ☐ 6 - 10 patients
- ☐ 11 - 15 patients
- ☐ More than 15 patients

---

4. What is your current position? (check only one)

Physician:

- ☐ Foundation doctor year 1 (FY1)
- ☐ Foundation doctor year 2 (FY2)
- ☐ Specialty registrar
- ☐ Consultant
- ☐ Other, please specify:____

---

5. How many years have you been practising as a physician? (check only one)

- ☐ 0 – 5 years
- ☐ 6 – 10 years
- ☐ 11 – 15 years
- ☐ 16 – 20 years
- ☐ 21 – 25 years
- ☐ 26 years or more

Please note that it is not permitted to go back and rectify previous questions.
Four scenarios now follow, illustrating typical clinical situations in the emergency department.

Scenario 1

An otherwise healthy 18-year-old girl is hospitalised on suspicion of meningitis. She has thrown up and has diarrhoea. On examination, she appears pale with cold skin, a slightly increased heart rate, normal blood pressure, and with decreased level of consciousness (Glasgow Coma Scale (GSC) score of 14). Laboratory tests show:

- pH 7.4 (ref.: 7.35 – 7.45);
- Plasma (P)-glucose 5.0 mmol/L (ref.: 4.2 – 6.3 mmol/l);
- P-[Na^+] 138 mmol/L (ref.: 137-145 mmol/L);
- P-(K^+] 3.6 mmol/L (ref.: 3.5 – 4.4 mmol/L).

The patient is unable to take fluids per os.

6. Which of the following is your first-line treatment of choice for this patient? (check only one)

☐ **Darrow–glucose**
(1 L contains 31 mmol sodium (1 g sodium chloride and 1.56 g sodium lactate), 55 g glucose monohydrate (278 mmol glucose), 0.67 g potassium chloride)

X **Isotonic saline**[1]
(1 L contains 9 g (154 mmol) sodium chloride)

☐ **Ringer's acetate**
(1 L contains 130 mmol sodium (4.1 g sodium acetate and 5.9 g sodium chloride), 0.295 g calcium chloride, 0.3 g potassium chloride, 0.2 g magnesium chloride)

☐ **3% NaCl**
(1 L contains 30 g (513 mmol) sodium chloride)

☐ **Potassium–sodium–glucose**
(1 L contains 2.338 g sodium chloride (40 mmol), 55 g (278 mmol) glucose (as monohydrate), and 1.492 g potassium chloride)

☐ **0.9% NaCl with 5% glucose**
(1 L contains 50 g (278 mmol) glucose and 9 g (154 mmol) sodium chloride)

☐ **0.45% sodium chloride with 2.5% glucose isotonic**
(1 L contains 25 g (139 mmol) water free glucose and 4.5 g sodium chloride (77 mmol))

☐ **Glucose 5% isotonic**
(1 L contains 55 g (278 mmol) glucose)

☐ **Do not know**

---

[1] Isotonic saline is recommended in patients with increased intracranial pressure [1–3].

Scenario 2

A 5-year-old boy arrives at the emergency department with a head injury after falling from a bike. He is experiencing headache and nausea, but no vomiting or signs of hypovolaemia. He has been unconscious for half an hour; however, the CT scan, clinical examination and laboratory results are all normal.

- pH 7.4 (ref.: 7.35 – 7.45);
- Plasma (P)-glukose 5.8 mmol/L (ref.: 4.2 – 6.3 mmol/l);
- P-[Na^+] 138 mmol/L (ref.: 137-145 mmol/L);
- P-[K^+] 3.6 mmol/L (ref.: 3.5 – 4.4 mmol/L).

Due to nausea, the patient is unable to take fluids per os.

7. Which of the following is your first-line treatment of choice for this patient? (check only one)

☐ **Potassium–sodium–glucose**
(1 L contains 2.338 g sodium chloride (40 mmol), 55 g (278 mmol) glucose (as monohydrate), and 1.492 g potassium chloride)

☐ **3% NaCl**
(1 L contains 30 g (513 mmol) sodium chloride)

☐ **Ringer's acetate**
(1 L contains 130 mmol sodium (4.1 g sodium acetate and 5.9 g sodium chloride), 0.295 g calcium chloride, 0.3 g potassium chloride, 0.2 g magnesium chloride)

☐ **0.45% sodium chloride with 2.5% glucose isotonic**
(1 L contains 25 g (139 mmol) water free glucose and 4.5 g sodium chloride (77 mmol))

☐ **Darrow–glucose**
(1 L contains 31 mmol sodium (1 g sodium chloride and 1.56 g sodium lactate), 55 g glucose monohydrate (278 mmol glucose), 0.67 g potassium chloride)

☐ **Isotonic saline**
(1 L contains 9 g (154 mmol) sodium chloride)

X **0.9% NaCl with 5% glucose**[2]
(1 L contains 50 g (278 mmol) glucose and 9 g (154 mmol) sodium chloride)

☐ **Glucose 5% isotonic**
(1 L contains 55 g (278 mmol) glucose)

☐ **Do not know**

---

[2] Isotonic saline with glucose is recommended for maintenance treatment in children [4,5].

Scenario 3

A 75-year-old woman arrives at the emergency department with a hip fracture after a fall. There are no signs of head injury. The patient has had a poor appetite for a long time. Medical history includes thiazide diuretics for hypertension, but otherwise she is healthy. Clinical examination shows symptoms of hypovolaemia: cold and pale skin, heart rate at 100 bpm, and a slightly increased respiratory rate. Laboratory findings:

- pH 7.4 (ref.: 7.35 – 7.45);
- Plasma (P)-glukose 6 mmol/L (ref.: 4.2 – 6.3 mmol/l);
- P-[Na^+] 110 mmol/L (ref.: 137 - 145 mmol/L);
- P-[K^+] 3.6 mmol/L (ref.: 3.5 – 4.4 mmol/L).

8. Which of the following is your first-line treatment of choice for this patient? (check only one)

☐ **Darrow–glucose**
(1 L contains 31 mmol sodium (1 g sodium chloride and 1.56 g sodium lactate), 55 g glucose monohydrate (278 mmol glucose), 0.67 g potassium chloride)

X **Isotonic saline**[3]
(1 L contains 9 g (154 mmol) sodium chloride)

☐ **3% NaCl**
(1 L contains 30 g (513 mmol) sodium chloride)

☐ **Potassium–sodium–glucose**
(1 L contains 2.338 g sodium chloride (40 mmol), 55 g (278 mmol) glucose (as monohydrate), and 1.492 g potassium chloride)

☐ **0.9% NaCl with 5% glucose**
(1 L contains 50 g (278 mmol) glucose and 9 g (154 mmol) sodium chloride)

☐ **0.45% sodium chloride with 2.5% glucose isotonic**
(1 L contains 25 g (139 mmol) water free glucose and 4.5 g sodium chloride (77 mmol))

☐ **Glucose 5% isotonic**
(1 L contains 55 g (278 mmol) glucose)

X **Ringer's acetate**
(1 L contains 130 mmol sodium (4.1 g sodium acetate and 5.9 g sodium chloride), 0.295 g calcium chloride, 0.3 g potassium chloride, 0.2 g magnesium chloride)

☐ **Do not know**

---

[3] Either isotonic saline or Ringers is recommended in hypovolaemic patients with asymptomatic hyponatraemia [1,6].

Scenario 4

A 28-year-old woman is hospitalised on suspicion of medication poisoning and large intake of water. She vomits and complains about headaches. She exhibits strange behaviour, has muscle rigidity and a Glasgow Coma Scale (GSC) score of 14. ABC is normal. Arterial blood gas shows:

- pH 7.48 (ref.: 7.35 – 7.45);
- Plasma (P)-glukose 5.4 mmol/L (ref.: 4.2 – 6.3 mmol/l);
- P-[Na^+] 118 mmol/L (ref.: 137 - 145 mmol/L);
- P-[K^+] 3.6 mmol/L (ref.: 3.5 – 4.4 mmol/L);
- pO_2 14 kPa (ref.: 11.1 – 14.4 kPa);
- pCO_2 4 kPa (ref.: 4.3 – 5.7 kPa).

9. Which of the following is your first-line treatment of choice for this patient? (check only one)

☐ **Potassium–sodium–glucose**
(1 L contains 2.338 g sodium chloride (40 mmol), 55 g (278 mmol) glucose (as monohydrate), and 1.492 g potassium chloride)

☐ **Isotonic saline**
(1 L contains 9 g (154 mmol) sodium chloride)

☐ **Ringer's acetate**
(1 L contains 130 mmol sodium (4.1 g sodium acetate and 5.9 g sodium chloride), 0.295 g calcium chloride, 0.3 g potassium chloride, 0.2 g magnesium chloride)

X **3% NaCl**[4]
(1 L contains 30 g (513 mmol) sodium chloride)

☐ **0.9% NaCl with 5% glucose**
(1 L contains 50 g (278 mmol) glucose and 9 g (154 mmol) sodium chloride)

☐ **Darrow–glucose**
(1 L contains 31 mmol sodium (1 g sodium chloride and 1.56 g sodium lactate), 55 g glucose monohydrate (278 mmol glucose), 0.67 g potassium chloride)

☐ **0.45% sodium chloride with 2.5% glucose isotonic**
(1 L contains 25 g (139 mmol) water free glucose and 4.5 g sodium chloride (77 mmol))

☐ **Glucose 5% isotonic**
(1 L contains 55 g (278 mmol) glucose)

☐ **Do not know**

---

[4] Hypertonic (3%) saline is recommended for patients with symptomatic hyponatraemia (e.g. altered level of consciousness (DK), vomiting (EU), polydipsia (US), headache (UpToDate)) [1,7–9].

Renal water excretion

Imagine an acutely ill patient in need of IV fluid.

10. Which of the following sentences is correct? (check only one)

- ☐ Most often acutely ill patients in need of IV fluids have increased renal water excretion
- X[5] Most often acutely ill patients in need of IV fluids have decreased renal water excretion
- ☐ Most often acutely ill patients in need of IV fluids have normal renal water excretion
- ☐ Do not know

[5] Renal water excretion is often reduced in acutely ill patients [10].

IV fluids impact on plasma sodium concentration (P-Na)

Four questions now follow for which you should imagine a patient at risk of reduced renal water excretion, and in need of a larger amount (> 20 ml/kg) of different IV fluids.

11. What is the most likely way that the following IV fluids will affect the plasma sodium concentration (P-Na) in such a patient?

    Please tick one box only for each IV fluid

	Large increase in P-Na with a risk of sodium overload	Slight increase in P-Na	Un-changed	Slight decrease in P-Na	Large decrease in P-Na with a risk of hyponatraemia	Do not know
**Ringer's lactate**[6] (1 L contains 130 mmol sodium)				X		
**Darrow–glucose** (1 L contains 31 mmol sodium)					X	
**Potassium–sodium–glucose** (1 L contains 40 mmol)					X	
**0.9% NaCl and 5% glucose** (1 L contains 154 mmol sodium chloride)			X			

[6] Ringer's lactate is hypotonic because it has a lower sodium concentration than extracellular fluid [1,11].

Hyperglycaemia and plasma sodium

12. Which one of the following sentences are correct? (check only one)

- X In case of increased blood sugar (above 12 mmol/L), the measured plasma sodium (P-Na) must be corrected because the measured P-Na is "falsely low".[7]
- ☐ In case of increased blood sugar (above 12 mmol/L), the measured plasma sodium (P-Na) must be corrected because the measured P-Na is "falsely high".
- ☐ In case of increased blood sugar (above 12 mmol/L), there is no reason to correct the measured plasma sodium (P-Na) because the measured P-Na is correct.
- ☐ Do not know.

---

[7] The measured plasma sodium is falsely low and must be corrected in the presence of hyperglycaemia [1,7].

Patients at high risk of severe symptoms upon inappropriate IV fluid treatment

13. Which of these diseases/symptoms may be indicative of potentially increased intracranial pressure (ICP) and therefore require special attention in relation to fluid treatment since even minor changes in plasma sodium may be critical? (check only one for each symptom/disease)

	**Yes**	**No**	**Do not know**
Meningitis[8]	x		
	Yes	No	Do not know
Shortness of breath		x	
	Yes	No	Do not know
Concussion	x		
	Yes	No	Do not know
Chest pain		x	
	Yes	No	Do not know
Seizure	x		
	Yes	No	Do not know
Acute liver failure	x		
	Yes	No	Do not know
Acute abdomen		x	
	Yes	No	Do not know
Hip fracture		x	

[8] Meningitis, concussion, seizure and acute liver failure are all causes of increased intracranial pressure [2].

Severe symptoms of hyponatraemia

14. Which of the following symptoms are indicative of severe symptoms of hyponatraemia and require acute treatment of hyponatraemia? (check only one for each symptom)

	**Yes**	**No**	**Do not know**
Altered level of consciousness [9]	x		
	Yes	No	Do not know
Seizure	x		
	Yes	No	Do not know
Infection		x	
	Yes	No	Do not know
Chest pain		x	
	Yes	No	Do not know
Muscle rigidity	x		
	Yes	No	Do not know
Anaemia		x	

---

[9] Examples of severe symptoms of hyponatraemia vary across guidelines. According to the Danish Endocrine Society altered level of consciousness, seizures and muscle rigidity are severe symptoms of hyponatraemia [1].

Prevention and treatment of overcorrection

Imagine a patient at high risk of brain damage (e.g., P-Na <115, PK < 2.5, malnutrition and/or severe liver disease) due to osmotic demyelination.

15. a) What is the maximum correction of plasma sodium per day for such a patient? (check only one)

X 6 mmol/l[10]

☐ 8 mmol/l

☐ 16 mmol/l

☐ 20 mmol/l

☐ Do not know

Imagine a patient without a high risk of brain damage due to osmotic demyelination.

15. b) What is the maximum correction of plasma sodium per day for such a patient? (check only one)

☐ 6 mmol/l

X 8 mmol/l

☐ 16 mmol/l

☐ 20 mmol/l

☐ Do not know

---

[10] The recommended limits for P-Na correction vary across guidelines. The Danish Endocrine Society and UpToDate recommend 6-8 mmol/L depending on the patient's symptoms [1,12].

In relation to IV fluid treatment for hyponatraemia, there is a risk that plasma sodium levels will rise too rapidly and exceed the maximum recommended limit of plasma sodium.

16. How would you prevent plasma sodium from rising too rapidly in relation to IV fluid treatment of hyponatraemia, and thus exceeding the recommended limits for plasma sodium (P-Na) correction? (select all that apply)

X I record the administration of IV fluids

☐ I recommend fluid restriction

X I record the first low P-Na level

X I monitor P-Na regularly

☐ I administer 5% glucose

X I record the maximum recommended increase of P-Na

☐ I encourage the patient to drink water

☐ Do not know

---

17. Which of the following would you administer to a patient whose plasma sodium concentration raise too rapidly? (select all that apply)

X Water per os[11]

☐ Fluid restriction (fluid intake is limited to less than 1 L/day)

☐ Isotonic saline (1 L contains 9 g (154 mmol) sodium chloride)

☐ Ringer's acetate (1 L contains 130 mmol sodium (4.1 g sodium acetate and 5.9 g sodium chloride), 0.295 g calcium chloride, 0.3 g potassium chloride, 0.2 g magnesium chloride)

☐ 3% NaCl (1 L contains 30 g (513 mmol) sodium chloride)

X Glucose 5% isotonic (1 L contains 55 g (278 mmol) glucose)

☐ Do not know

---

[11] Water per os or 5% glucose is recommended for patients in whom plasma sodium concentration rises too rapidly [1,7,8,12].

18. What is the most common cause of over-correction? (check only one)

X Large diuresis[12]

- ☐ Increased sodium secretion
- ☐ Increased water intake
- ☐ Inadequate water intake
- ☐ Increased sodium intake
- ☐ Reduced renal water excretion

[12] Large diuresis is recognised as a common cause of over-correction in the guidelines set by the Danish Endocrine Society and Sterns et al. [1,13].

Final questions

19. Did you use any aids to complete the questionnaire?

- ☐ No
- ☐ Yes

If yes, which did you use? (select all that apply)

- ☐ The treatment guideline of the department
- ☐ Pro.medicin.dk
- ☐ The guideline on hyponatraemia from the Association of Clinical Endocrinologists
- ☐ Other:____

Thank you very much for your participation. If you have any comments to the questionnaire or research project, you can write them below or send an email to Per Sindahl:

References

1. Dansk Endokrinologisk Selskab Hyponatriæmi [National Treatment Guideline Endocrinology: Hyponatraemia]. 2019. Available online: https://endocrinology.dk/nbv/andre-endokrinologiske-sygdomme/hyponatriaemi/ (accessed on 26 January 2022).

2. Evaluation and management of elevated intracranial pressure in adults - UpToDate [Internet]. [cited 2020 Apr 5]. Available from: https://www.uptodate.com/contents/evaluation-and-management-of-elevated-intracranial-pressure-in-adults?search=icp%20management&source=search_result&selectedTitle=1~150&usage_type=default&display_rank=1

3. Pharmacovigilance Risk Assessment Committee. Recommendations on signals adopted at the 3-6 July 2017 PRAC [Internet]. 2017. Available from: https://www.ema.europa.eu/en/committees/prac/prac-agendas-minutes-highlights#minutes-section

4. Feld LG, Neuspiel DR, Foster BA, Leu MG, Garber MD, Austin K, et al. Clinical Practice Guideline: Maintenance Intravenous Fluids in Children. Pediatrics. 2018;142.

5. Maintenance intravenous fluid therapy in children - UpToDate [Internet]. [cited 2020 Aug 12]. Available from: https://www.uptodate.com/contents/maintenance-intravenous-fluid-therapy-in-children?search=maintenance%20fluids%20children&source=search_result&selectedTitle=1~150&usage_type=default&display_rank=1

6. Liamis G, Filippatos TD, Elisaf MS. Correction of hypovolemia with crystalloid fluids: Individualizing infusion therapy. Postgrad Med. 2015;127:405–12.

7. Spasovski G, Vanholder R, Allolio B, Annane D, Ball S, Bichet D, et al. Clinical practice guideline on diagnosis and treatment of hyponatraemia. Intensive Care Med. 2014;40:320–31.

8. Verbalis JG, Goldsmith SR, Greenberg A, Korzelius C, Schrier RW, Sterns RH, et al. Diagnosis, Evaluation, and Treatment of Hyponatremia: Expert Panel Recommendations. Am J Med. 2013;126:S1–42.

9. Overview of the treatment of hyponatremia in adults - UpToDate [Internet]. [cited 2020 Aug 11]. Available from: https://www.uptodate.com/contents/overview-of-the-treatment-of-hyponatremia-in-adults?search=hyponatremia&source=search_result&selectedTitle=1~150&usage_type=default&display_rank=1#H2818747047

10. Moritz ML, Ayus JC. Maintenance Intravenous Fluids in Acutely Ill Patients. N Engl J Med. 2015;373:1350–60.

11. Myburgh JA, Mythen MG. Resuscitation Fluids. N Engl J Med. Massachusetts Medical Society; 2013;369:1243–51.

12. Osmotic demyelination syndrome (ODS) and overly rapid correction of hyponatremia - UpToDate [Internet]. [cited 2020 Aug 12]. Available from: https://www.uptodate.com/contents/osmotic-demyelination-syndrome-ods-and-overly-rapid-correction-of-hyponatremia?search=hyponatremia%20treatment&source=search_result&selectedTitle=7~150&usage_type=default&display_rank=5

13. Sterns RH, Nigwekar SU, Hix JK. The Treatment of Hyponatremia. Semin Nephrol. 2009;29:282–99.

Post-intervention questionnaire

[A cross (X) is used to indicate correct answers. References were not part of the questionnaire presented to the participants].

Background questions

5. **What is your gender?**

   - ☐ Female
   - ☐ Male
   - ☐ Other

---

6. **Which of the following categories describe your age?** (check only one)

   - ☐ 18 – 24 years
   - ☐ 25 – 34 years
   - ☐ 35 – 44 years
   - ☐ 45 – 54 years
   - ☐ 55 – 64 years
   - ☐ 65 years or older

---

7. **How many patients do you treat with IV fluids in an average week?** (check only one)

   - ☐ 0 patients
   - ☐ 1 - 5 patients
   - ☐ 6 - 10 patients
   - ☐ 11 - 15 patients
   - ☐ More than 15 patients

---

8. **What is your current position?** (check only one)

- ☐ Foundation doctor year 1 (FY1)
- ☐ Foundation doctor year 2 (FY2)
- ☐ Specialty registrar
- ☐ Consultant
- ☐ Other, please specify:____

---

6. How many years have you been practising as a physician? (check only one)

- ☐ 0 – 5 years
- ☐ 6 – 10 years
- ☐ 11 – 15 years
- ☐ 16 – 20 years
- ☐ 21 – 25 years
- ☐ 26 years or more

---

7. What is your primary medical specialty? (check only one)

- ☐ General practitioner
- ☐ Paediatrics
- ☐ Surgery
- ☐ Orthopaedic surgery
- ☐ Anaesthesiology
- ☐ Emergency medicine
- ☐ Other: _______________________________

Please note that it is not permitted to go back and rectify previous questions from this point onwards. Four scenarios now follow, illustrating typical clinical situations in the emergency department.

Scenario 1

An otherwise-healthy 18-year-old girl is hospitalised on suspicion of meningitis. She has thrown up and has diarrhoea. On examination, she appears pale with cold skin, a slightly increased heart rate, normal blood pressure, and with decreased level of consciousness (Glasgow Coma Scale (GSC) score of 14). Laboratory tests show:

- pH 7.4 (ref.: 7.35 – 7.45);
- Plasma (P)-glucose 5.0 mmol/L (ref.: 4.2 – 6.3 mmol/l);
- P-[Na^+] 138 mmol/L (ref.: 137-145 mmol/L);
- P-[K^+] 3.6 mmol/L (ref.: 3.5 – 4.4 mmol/L).

The patient is unable to take fluids per os.

8. **Which of the following options is your first-line treatment of choice for this patient?** (check only one)

☐ **Darrow–glucose**
(1 L contains 31 mmol sodium (1 g sodium chloride and 1.56 g sodium lactate), 55 g glucose monohydrate (278 mmol glucose), 0.67 g potassium chloride)

X **Isotonic saline**[13]
(1 L contains 9 g (154 mmol) sodium chloride)

☐ **Ringer's acetate**
(1 L contains 130 mmol sodium (4.1 g sodium acetate and 5.9 g sodium chloride), 0.295 g calcium chloride, 0.3 g potassium chloride, 0.2 g magnesium chloride)

☐ **3% NaCl**
(1 L contains 30 g (513 mmol) sodium chloride)

☐ **Potassium–sodium–glucose**
(1 L contains 2.338 g sodium chloride (40 mmol), 55 g (278 mmol) glucose (as monohydrate), and 1.492 g potassium chloride)

☐ **0.9% NaCl with 5% glucose**
(1 L contains 50 g (278 mmol) glucose and 9 g (154 mmol) sodium chloride)

☐ **0.45% sodium chloride with 2.5% glucose isotonic**
(1 L contains 25 g (139 mmol) water free glucose and 4.5 g sodium chloride (77 mmol))

☐ **Glucose 5% isotonic**
(1 L contains 55 g (278 mmol) glucose)

☐ **Do not know**

---

[13] Isotonic saline is recommended in patients with increased intracranial pressure [1–3].

Scenario 2

A 5-year-old boy arrives at the emergency department with a head injury after falling from a bike. He has a headache and is experiencing nausea, but no vomiting or signs of hypovolaemia. He has been unconscious for half an hour; however, the CT scan, clinical examination and laboratory results are all normal.

- pH 7.4 (ref.: 7.35 – 7.45);
- Plasma (P)-glukose 5.8 mmol/L (ref.: 4.2 – 6.3 mmol/l);
- P-[Na^+] 138 mmol/L (ref.: 137-145 mmol/L);
- P-[K^+] 3.6 mmol/L (ref.: 3.5 – 4.4 mmol/L).

Due to nausea, the patient is unable to take fluids per os.

9. **Which of the following options is your first-line treatment of choice for this patient?** (check only one)

☐ **Potassium–sodium–glucose**
(1 L contains 2.338 g sodium chloride (40 mmol), 55 g (278 mmol) glucose (as monohydrate), and 1.492 g potassium chloride)

☐ **3% NaCl**
(1 L contains 30 g (513 mmol) sodium chloride)

☐ **Ringer's acetate**
(1 L contains 130 mmol sodium (4.1 g sodium acetate and 5.9 g sodium chloride), 0.295 g calcium chloride, 0.3 g potassium chloride, 0.2 g magnesium chloride)

☐ **0.45% sodium chloride with 2.5% glucose isotonic**
(1 L contains 25 g (139 mmol) water free glucose and 4.5 g sodium chloride (77 mmol))

☐ **Darrow–glucose**
(1 L contains 31 mmol sodium (1 g sodium chloride and 1.56 g sodium lactate), 55 g glucose monohydrate (278 mmol glucose), 0.67 g potassium chloride)

☐ **Isotonic saline**
(1 L contains 9 g (154 mmol) sodium chloride)

X **0.9% NaCl with 5% glucose**[14]
(1 L contains 50 g (278 mmol) glucose and 9 g (154 mmol) sodium chloride)

☐ **Glucose 5% isotonic**
(1 L contains 55 g (278 mmol) glucose)

☐ **Do not know**

---

[14] Isotonic saline with glucose is recommended for maintenance treatment in children [4,5].

Scenario 3

A 75-year-old woman arrives at the emergency department with a hip fracture after a fall. There are no signs of head injury. The patient has had a poor appetite for a long time. Medical history includes thiazide diuretics for hypertension, but otherwise she is healthy. Clinical examination shows symptoms of hypovolaemia: cold and pale skin, heart rate at 100 bpm, and a slightly increased respiratory rate. Laboratory findings:

- pH 7.4 (ref.: 7.35 – 7.45);
- Plasma (P)-glucose 6 mmol/L (ref.: 4.2 – 6.3 mmol/l);
- P-[Na^+] 110 mmol/L (ref.: 137 - 145 mmol/L);
- P-[K^+] 3.6 mmol/L (ref.: 3.5 – 4.4 mmol/L).

10. **Which of the following options is your first-line treatment of choice for this patient?** (check only one)

☐ **Darrow–glucose**
(1 L contains 31 mmol sodium (1 g sodium chloride and 1.56 g sodium lactate), 55 g glucose monohydrate (278 mmol glucose), 0.67 g potassium chloride)

X **Isotonic saline**[15]
(1 L contains 9 g (154 mmol) sodium chloride)

☐ **3% NaCl**
(1 L contains 30 g (513 mmol) sodium chloride)

☐ **Potassium–sodium–glucose**
(1 L contains 2.338 g sodium chloride (40 mmol), 55 g (278 mmol) glucose (as monohydrate), and 1.492 g potassium chloride)

☐ **0.9% NaCl with 5% glucose**
(1 L contains 50 g (278 mmol) glucose and 9 g (154 mmol) sodium chloride)

☐ **0.45% sodium chloride with 2.5% glucose isotonic**
(1 L contains 25 g (139 mmol) water free glucose and 4.5 g sodium chloride (77 mmol))

☐ **Glucose 5% isotonic**
(1 L contains 55 g (278 mmol) glucose)

X **Ringer's acetate**
(1 L contains 130 mmol sodium (4.1 g sodium acetate and 5.9 g sodium chloride), 0.295 g calcium chloride, 0.3 g potassium chloride, 0.2 g magnesium chloride)

☐ **Do not know**

[15] Either isotonic saline or Ringers is recommended in hypovolaemic patients with asymptomatic hyponatraemia [1,6].

Scenario 4

A 28-year-old woman is hospitalised on suspicion of medication poisoning and large intake of water. She vomits and complains about headaches. She exhibits strange behaviour, has muscle rigidity and a Glasgow Coma Scale (GSC) score of 14. ABC is normal. Laboratory findings show:

- pH 7.48 (ref.: 7.35 – 7.45);
- Plasma (P)-glucose 5.4 mmol/L (ref.: 4.2 – 6.3 mmol/l);
- P-[Na^+] 118 mmol/L (ref.: 137 - 145 mmol/L);
- P-[K^+] 3.6 mmol/L (ref.: 3.5 – 4.4 mmol/L);
- pO_2 14 kPa (ref.: 11.1 – 14.4 kPa);
- pCO_2 4 kPa (ref.: 4.3 – 5.7 kPa).

11. **Which of the following options is your first-line treatment of choice for this patient?** (check only one)

☐ **Potassium–sodium–glucose**
(1 L contains 2.338 g sodium chloride (40 mmol), 55 g (278 mmol) glucose (as monohydrate), and 1.492 g potassium chloride)

☐ **Isotonic saline**
(1 L contains 9 g (154 mmol) sodium chloride)

☐ **Ringer's acetate**
(1 L contains 130 mmol sodium (4.1 g sodium acetate and 5.9 g sodium chloride), 0.295 g calcium chloride, 0.3 g potassium chloride, 0.2 g magnesium chloride)

X **3% NaCl**[16]
(1 L contains 30 g (513 mmol) sodium chloride)

☐ **0.9% NaCl with 5% glucose**
(1 L contains 50 g (278 mmol) glucose and 9 g (154 mmol) sodium chloride)

☐ **Darrow–glucose**
(1 L contains 31 mmol sodium (1 g sodium chloride and 1.56 g sodium lactate), 55 g glucose monohydrate (278 mmol glucose), 0.67 g potassium chloride)

☐ **0.45% sodium chloride with 2.5% glucose isotonic**
(1 L contains 25 g (139 mmol) water free glucose and 4.5 g sodium chloride (77 mmol))

☐ **Glucose 5% isotonic**
(1 L contains 55 g (278 mmol) glucose)

☐ **Do not know**

---

[16] Hypertonic (3%) saline is recommended for patients with symptomatic hyponatraemia (e.g. altered level of consciousness (Danish guideline), vomiting (European guideline), polydipsia (US guideline), headache (UpToDate)) [1,7–9].

Renal water excretion

Imagine an acutely ill patient in need of IV fluid.

12. **Which of the following sentences are correct?** (check only one)

☐ Most often acutely ill patients in need of IV fluids have increased renal water excretion

X[17] Most often acutely ill patients in need of IV fluids have decreased renal water excretion

☐ Most often acutely ill patients in need of IV fluids have normal renal water excretion

☐ Do not know

---

[17] Renal water excretion is often reduced in acutely ill patients [10].

IV fluids impact on plasma sodium concentration (P-Na)

Four questions now follow for which you should imagine a patient at risk of reduced renal water excretion, and in need of a larger amount (> 20 ml/kg) of different IV fluids.

13. What is the most likely way that the following IV fluids will affect the plasma sodium concentration (P-Na) in such a patient?

Please tick one box only for each IV fluid

	Large increase in P-Na with a risk of sodium overload	Slight increase in P-Na	Un-changed	Slight decrease in P-Na	Large decrease in P-Na with a risk of hyponatraemia	Do not know
**Ringer's lactate**[18] (1 L contains 130 mmol sodium)				X		
**Darrow–glucose** (1 L contains 31 mmol sodium)					X	
**Potassium–sodium–glucose** (1 L contains 40 mmol)					X	
**0.9% NaCl and 5% glucose** (1 L contains 154 mmol sodium chloride)			X			

[18] Ringer's lactate is hypotonic because it have a lower sodium concentration than extracellular fluid [1,11].

Hyperglycaemia and plasma sodium

14. Which one of the following sentences is correct? (check only one)

X In case of increased blood sugar (above 12 mmol/L), the measured plasma sodium (P-Na) must be corrected because the measured P-Na is "falsely low".[19]

☐ In case of increased blood sugar (above 12 mmol/L), the measured plasma sodium (P-Na) must be corrected because the measured P-Na is "falsely high".

☐ In case of increased blood sugar (above 12 mmol/L), there is no reason to correct the measured plasma sodium (P-Na) because the measured P-Na is correct.

☐ Do not know.

---

[19] The measured plasma sodium is falsely low and must be corrected in the presence of hyperglycaemia [1,7].

Patients at high risk of severe symptoms upon inappropriate IV fluid treatment

15. **Which of these diseases/symptoms may be indicative of potentially increased intracranial pressure (ICP) and therefore require special attention in relation to fluid treatment since even minor changes in plasma sodium may be critical?** (check only one for each symptom/disease)

	**Yes**	**No**	**Do not know**
Meningitis[20]	x		
	Yes	No	Do not know
Shortness of breath		x	
	Yes	No	Do not know
Concussion	x		
	Yes	No	Do not know
Chest pain		x	
	Yes	No	Do not know
Seizure	x		
	Yes	No	Do not know
Acute liver failure	x		
	Yes	No	Do not know
Acute abdomen		x	
	Yes	No	Do not know
Hip fracture		x	

[20] Meningitis, concussion, seizure and acute liver failure are all causes of increased intracranial pressure [2].

Severe symptoms of hyponatraemia

16. **Which of the following symptoms are indicative of severe symptoms of hyponatraemia and require acute treatment of hyponatraemia?** (check only one for each symptom)

	**Yes**	**No**	**Do not know**
Altered level of consciousness [21]	x		
	Yes	No	Do not know
Seizure	x		
	Yes	No	Do not know
Infection		x	
	Yes	No	Do not know
Chest pain		x	
	Yes	No	Do not know
Muscle rigidity	x		
	Yes	No	Do not know
Anaemia		x	

---

[21] Examples of severe symptoms of hyponatraemia vary across guidelines. According to the Danish Endocrine Society altered level of consciousness, seizures and muscle rigidity are severe symptoms of hyponatraemia [1].

Sodium folder – A guide to IV fluid treatment

Below you see the pocket-folder “Sodium folder—A guide to IV fluid treatment”, which was distributed to all emergency departments in June 2020.

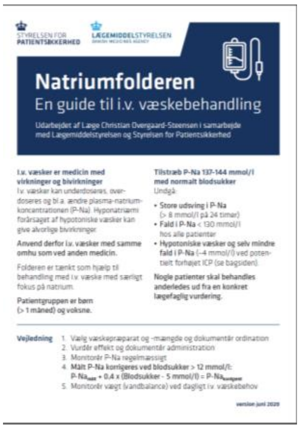

STYRELSEN FOR PATIENTSIKKERHED

LÆGEMIDDELSTYRELSEN DANISH MEDICINES AGENCY

Natriumfolderen

En guide til i.v. væskebehandling

Udarbejdet af Læge Christian Overgaard-Steensen i samarbejde med Lægemiddelstyrelsen og Styrelsen for Patientsikkerhed

I.v. væsker er medicin med virkninger og bivirkninger
I.v. væsker kan underdoseres, overdoseres og bl.a. ændre plasma-natrium-koncentrationen (P-Na). Hyponatriæmi forårsaget af hypotoniske væsker kan give alvorlige bivirkninger.

Anvend derfor i.v. væsker med samme omhu som ved anden medicin.

Folderen er tænkt som hjælp til behandling med i.v. væske med særligt fokus på natrium.

Patientgruppen er børn (> 1 måned) og voksne.

Tilstræb P-Na 137-144 mmol/l med normalt blodsukker
Undgå:

- **Store udsving i P-Na** (> 8 mmol/l på 24 timer)
- **Fald i P-Na** < 130 mmol/l hos alle patienter
- **Hypotoniske væsker og selv mindre fald i P-Na** (~4 mmol/l) ved potentielt forhøjet ICP (se bagsiden)

Nogle patienter skal behandles anderledes ud fra en konkret lægefaglig vurdering.

Vejledning

1. Vælg væskepræparat og -mængde og dokumentér ordination
2. Vurdér effekt og dokumentér administration
3. Monitorér P-Na regelmæssigt
4. **Målt P-Na korrigeres ved blodsukker > 12 mmol/l:** P-Na[illegible] + 0,4 x (Blodsukker - 5 mmol/l) = P-Na[illegible]
5. Monitorér vægt (vandbalance) ved dagligt i.v. væskebehov

version juni 2020

17. Have you received the above pictured pocket-folder “Sodium folder—A guide to IV fluid treatment”?

☐ Yes

☐ No

☐ I don’t remember receiving it/not sure

18. Did you read the pocket-folder?

☐ Yes

☐ No

☐ I don’t remember reading it/not sure

This question should only be answered if you have received and read the pocket-folder.

19. Please indicate your degree of agreement with the following two statements by placing a tick in the appropriate box

	Strongly agree	Agree	Neither agree nor disagree (neutral)	Disagree	Strongly disagree
a) I find the pocket-folder useful	☐	☐	☐	☐	☐

	Strongly agree	Agree	Neither agree nor disagree (neutral)	Disagree	Strongly disagree
b The pocket-folder has changed my prescribing behaviour patterns	☐	☐	☐	☐	☐

20. If you disagree or strongly disagree that the pocket-folder is useful, we would very much like to hear your opinion so we can improve the pocket-folder. You can write your email address below if we may contact you.

Mailing address: ____

Final questions

21. Did you use any aids to complete the questionnaire?

- ☐ No
- ☐ Yes

If yes, which did you use? (select all that apply)

- ☐ The treatment guideline of the department
- ☐ Pro.medicin.dk
- ☐ The guideline on hyponatraemia from the Association of Clinical Endocrinologists
- ☐ The pocket-folder "Sodium folder – A guide to IV fluid treatment"
- ☐ Other:______________________________

Thank you very much for your participation. If you have any comments to the questionnaire or research project, you can write them below or send an email to Per Sindahl, pesi@dkma.dk:

____
____
____
____
____
____

References

1. Dansk Endokrinologisk Selskab. NBV Endokrinologi: Hyponatriæmi [National Treatment Guideline Endocrinology: Hyponatraemia] [Internet]. 2016 [cited 2019 Sep 23]. Available from: http://www.endocrinology.dk/index.php/6-andre-endokrinologiske-sygdomme/2-hyponatriaemi

2. Evaluation and management of elevated intracranial pressure in adults - UpToDate [Internet]. [cited 2020 Apr 5]. Available from: https://www.uptodate.com/contents/evaluation-and-management-of-elevated-intracranial-pressure-in-adults?search=icp%20management&source=search_result&selectedTitle=1~150&usage_type=default&display_rank=1

3. Pharmacovigilance Risk Assessment Committee. Recommendations on signals adopted at the 3-6 July 2017 PRAC [Internet]. 2017. Available from: https://www.ema.europa.eu/en/committees/prac/prac-agendas-minutes-highlights#minutes-section

4. Feld LG, Neuspiel DR, Foster BA, Leu MG, Garber MD, Austin K, et al. Clinical Practice Guideline: Maintenance Intravenous Fluids in Children. Pediatrics. 2018;142.

5. Maintenance intravenous fluid therapy in children - UpToDate [Internet]. [cited 2020 Aug 12]. Available from: https://www.uptodate.com/contents/maintenance-intravenous-fluid-therapy-in-children?search=maintenance%20fluids%20children&source=search_result&selectedTitle=1~150&usage_type=default&display_rank=1

6. Liamis G, Filippatos TD, Elisaf MS. Correction of hypovolemia with crystalloid fluids: Individualizing infusion therapy. Postgrad Med. 2015;127:405–12.

7. Spasovski G, Vanholder R, Allolio B, Annane D, Ball S, Bichet D, et al. Clinical practice guideline on diagnosis and treatment of hyponatraemia. Intensive Care Med. 2014;40:320–31.

8. Verbalis JG, Goldsmith SR, Greenberg A, Korzelius C, Schrier RW, Sterns RH, et al. Diagnosis, Evaluation, and Treatment of Hyponatremia: Expert Panel Recommendations. Am J Med. 2013;126:S1–42.

9. Overview of the treatment of hyponatremia in adults - UpToDate [Internet]. [cited 2020 Aug 11]. Available from: https://www.uptodate.com/contents/overview-of-the-treatment-of-hyponatremia-in-adults?search=hyponatremia&source=search_result&selectedTitle=1~150&usage_type=default&display_rank=1#H2818747047

10. Moritz ML, Ayus JC. Maintenance Intravenous Fluids in Acutely Ill Patients. N Engl J Med. 2015;373:1350–60.

11. Myburgh JA, Mythen MG. Resuscitation Fluids. N Engl J Med. 2013;369:1243–51.

12. Osmotic demyelination syndrome (ODS) and overly rapid correction of hyponatremia - UpToDate [Internet]. [cited 2020 Aug 12]. Available from: https://www.uptodate.com/contents/osmotic-demyelination-syndrome-ods-and-overly-rapid-correction-of-hyponatremia?search=hyponatremia%20treatment&source=search_result&selectedTitle=7~150&usage_type=default&display_rank=5

13. Sterns RH, Nigwekar SU, Hix JK. The Treatment of Hyponatremia. Semin Nephrol. 2009;29:282–99.

Survey response

	Approached/invited/targeted* (n)	Started on questionnaire (n)	Responded to one or more scenarios (n)	Responded to all scenarios (n)	Completed all questions (n)	Response rate (%)
Pre	363	223	215	201	159	55
Post	363	206	180	154	130	42

*Estimated source population (i.e., physicians working at emergency departments in Denmark) from 2014.

Univariate analysis of background characteristics

Table S1: Univariate analysis of background characteristics for scenario 1

Variable		Prescribing practice of respondents from hospitals that participated both in the pre-intervention and post-intervention survey (N = 255)					
		Correct		Incorrect		Univariate Odds Ratio of incorrect responses (95% CI)	*p*-value
		n	%	*n*	%		
**Pre- or post-intervention**							
	Pre	129	66.2%	35	58.3%	Ref.	0.3
	Post	66	33.8%	25	41.7%	1.4 (0.8-2.5)	
**Gender***							
	Female	125	64.1%	27	45.0%	Ref.**	<0.1
	Male	70	35.9%	32	53.3%	2.1 (1.2-3.8)	
**Age**							
	18–34 years	64	32.8%	24	40.0%	Ref.	0.6
	35-44 years	61	31.3%	17	28.3%	0.7 (0.4-1.5)	
	≥45 years	70	35.9%	19	31.7%	0.7 (0.4-1.4)	
**Number of weekly treated patients with intravenous fluids** ***							
	0-5 patients	112	57.4%	17	28.3%	Ref.	<0.1
	> 5 patients	83	42.6%	41	68.3%	3.3 (1.7-6.1)	
**Years of practice** ***							
	≤5 years	61	31.3%	22	36.7%	Ref.	
	>5 years	132	67.7%	38	63.3%	0.8 (0.4-1.5)	
**Position** ****							
	Junior	100	51.3%	30	50.0%	Ref.	0.5
	Senior	95	48.7%	30	50.0%	1.1 (0.6-1.9)	
**Size**							
	Small/Medium	91	46.7%	38	63.3%	Ref.	<0.1
	Large	104	53.3%	22	36.7%	0.5 (0.3-0.9)	
**Complexity** *****							
	Medium	123	63.1%	50	83.3%	Ref.	<0.1
	High	72	36.9%	10	16.7%	0.3 (0.2-0.7)	
**Type** ******							
	Adult ED *******	46	23.6%	21	35.0%	Ref.	<0.1
	Pediatric ED	123	63.1%	16	26.7%	0.3 (0.1-0.6)	
	Combined general population ED ********	17	13.3%	20	38.3%	1.9 (0.9-4.2)	

* Gender = 'Other' not shown (n = 1). ** Ref = reference. *** Missing not shown (n = 2). **** Junior physicians, including foundation doctor year 1 and 2, specialty registrar and 'other' (medical students, unspecified junior doctor, pre-FY1, and PhD students). ***** Complexity, i.e., complexity of services they provide. ****** Combined general population emergency departments provide care for all patients in one area, while separate general population EDs provide care to children and adults in separate locations within a facility. ******* ED = emergency department. ******** This type also includes trauma centres.

Table S2: Univariate analysis of background characteristics for scenario 2

Variable		Correct *n*	Correct %	Incorrect *n*	Incorrect %	Univariate Odds Ratio of incorrect responses (95% CI)	*p*-value
		Prescribing practice of respondents from hospitals that participated both in the pre-intervention and post-intervention survey (N = 255)					
**Pre- or post-intervention**							
	Pre	110	61.1%	54	110	Ref.	
	Post	70	38.9%	21	70	0.6 (0.3-1.1)	0.1
**Gender***							
	Female	111	61.7%	41	54.7%	Ref.**	0.7
	Male	69	38.3%	33	44.0%	1.3 (0.7-2.2)	
**Age**							
	18–34 years	67	37.2%	21	28.0%	Ref.	<0.1
	35-44 years	59	32.8%	19	25.3%	1.0 (0.5-2.1)	
	≥45 years	54	30.0%	35	46.7%	2.1 (1.1-4.0)	
**Number of weekly treated patients with intravenous fluids** ***							
	0-5 patients	88	48.9%	41	54.7%	Ref.	0.5
	> 5 patients	91	50.6%	33	44.0%	0.8 (0.5-1.3)	
**Years of practice** ***							
	≤5 years	65	36.1%	18	24.0%	Ref.	0.2
	>5 years	114	63.3%	56	74.7%	1.8 (1.0-3.3)	
**Position** ****							
	Junior	99	55.0%	31	41.3%	Ref.	<0.1
	Senior	81	45.0%	44	58.7%	1.7 (1.0-3.0)	
**Size**							
	Small/Medium	86	47.8%	43	57.3%	Ref.	0.2
	Large	94	52.2%	32	42.7%	0.7 (0.4-1.2)	
**Complexity** *****							
	Medium	118	65.6%	55	73.3%	Ref.	0.2
	High	62	34.4%	20	26.7%	0.7 (0.4-1.3)	
**Type** ******							
	Adult ED *******	53	29.4%	14	18.7%	Ref.	<0.1
	Pediatric ED	90	50.0%	49	65.3%	2.1 (1.0-4.1)	
	Combined general population ED ********	37	20.6%	12	16.0%	1.2 (0.5-3.0)	

* Gender = 'Other' not shown (n = 1). ** Ref = reference. *** Missing not shown (n = 2). **** Junior physicians, including foundation doctor year 1 and 2, specialty registrar and 'other' (medical students, unspecified junior doctor, pre-FY1, and PhD students). ***** Complexity, i.e., complexity of services they provide. ****** Combined general population emergency departments provide care for all patients in one area, while separate general population EDs provide care to children and adults in separate locations within a facility. ******* ED = emergency department. ******** This type also includes trauma centres.

Table S3: Univariate analysis of background characteristics for scenario 3

Variable		Prescribing practice of respondents from hospitals that participated both in the pre-intervention and post-intervention survey (N = 255)					
		Correct		Incorrect		Univariate Odds Ratio of incorrect responses (95% CI)	*p*-value
		n	%	*n*	%		
**Pre- or post-intervention**							
	Pre	148	63.8%	16	148	Ref.	0.6
	Post	84	36.2%	7	84	0.8 (0.3-1.9)	
**Gender***							
	Female	141	60.8%	11	47.8%	Ref.**	0.5
	Male	90	38.8%	12	52.2%	1.7 (0.7-4.0)	
**Age**							
	18–34 years	80	34.5%	8	34.8%	Ref.	0.3
	35-44 years	68	29.3%	10	43.5%	1.5 (0.6-3.9)	
	≥45 years	84	36.2%	5	21.7%	0.6 (0.2-1.9)	
**Number of weekly treated patients with intravenous fluids** ***							
	0-5 patients	119	51.3%	10	43.5%	Ref.	0.8
	> 5 patients	111	47.8%	13	56.5%	1.4 (0.6-3.3)	
**Years of practice** ***							
	≤5 years	76	32.8%	7	30.4%	Ref.	0.8
	>5 years	154	66.4%	16	69.6%	1.1 (0.4-2.9)	
**Position** ****							
	Junior	118	50.9%	12	52.2%	Ref.	0.9
	Senior	114	49.1%	11	47.8%	0.9 (0.4-2.2)	
**Size**							
	Small/Medium	117	50.4%	12	52.2%	Ref.	0.9
	Large	115	49.6%	11	47.8%	0.9 (0.4-2.2)	
**Complexity** *****							
	Medium	155	66.8%	18	78.3%	Ref.	0.3
	High	77	33.2%	5	21.7%	0.6 (0.2-1.6)	
**Type** ******							
	Adult ED *******	62	26.7%	5	21.7%	Ref.	0.9
	Pediatric ED	126	54.3%	13	56.5%	1.3 (0.4-3.8)	
	Combined general population ED ********	44	19.0%	5	21.7%	1.4 (0.4-5.2)	

* Gender = 'Other' not shown (n = 1). ** Ref = reference. *** Missing not shown (n = 2). **** Junior physicians, including foundation doctor year 1 and 2, specialty registrar and 'other' (medical students, unspecified junior doctor, pre-FY1, and PhD students). ***** Complexity, i.e., complexity of services they provide. ****** Combined general population emergency departments provide care for all patients in one area, while separate general population EDs provide care to children and adults in separate locations within a facility. ******* ED = emergency department. ******** This type also includes trauma centres.

Table S4: Univariate analysis of background characteristics for scenario 4

Variable		Correct *n*	Correct %	Incorrect *n*	Incorrect %	Univariate Odds Ratio of incorrect responses (95% CI)	*p*-value
		Prescribing practice of respondents from hospitals that participated both in the pre-intervention and post-intervention survey (N = 255)					
**Pre- or post-intervention**							
	Pre	137	62.6%	27	75.0%	Ref.	
	Post	82	37.4%	9	25.0%	0.6 (0.2-1.2)	0.1
**Gender***							
	Female	130	59.4%	22	61.1%	Ref.**	1.0
	Male	88	40.2%	14	38.9%	0.9 (0.5-1.9)	
**Age**							
	18–34 years	74	33.8%	14	38.9%	Ref.	0.6
	35-44 years	66	30.1%	12	33.3%	1.0 (0.4-2.2)	
	≥45 years	79	36.1%	10	27.8%	0.7 (0.3-1.6)	
**Number of weekly treated patients with intravenous fluids** ***							
	0-5 patients	114	52.1%	15	41.7%	Ref.	0.5
	> 5 patients	103	47.0%	21	58.3%	1.6 (0.8-3.2)	
**Years of practice** ***							
	≤5 years	72	32.9%	11	30.6%	Ref.	0.8
	>5 years	145	66.2%	25	69.4%	1.1 (0.5-2.4)	
**Position** ****							
	Junior	110	50.2%	20	55.6%	Ref.	0.6
	Senior	109	49.8%	16	44.4%	0.8 (0.4-1.6)	
**Size**							
	Small/Medium	110	50.2%	19	52.8%	Ref.	0.8
	Large	109	49.8%	17	47.2%	0.9 (0.4-1.8)	
**Complexity** *****							
	Medium	152	69.4%	21	58.3%	Ref.	0.2
	High	67	30.6%	15	41.7%	1.6 (0.8-3.3)	
**Type** ******							
	Adult ED *******	54	24.7%	13	36.1%	Ref.	0.3
	Pediatric ED	123	56.2%	16	44.4%	0.5 (0.2-1.2)	
	Combined general population ED ********	42	19.2%	7	19.4%	0.7 (0.3-1.9)	

* Gender = 'Other' not shown (n = 1). ** Ref = reference. *** Missing not shown (n = 2). **** Junior physicians, including foundation doctor year 1 and 2, specialty registrar and 'other' (medical students, unspecified junior doctor, pre-FY1, and PhD students). ***** Complexity, i.e., complexity of services they provide. ****** Combined general population emergency departments provide care for all patients in one area, while separate general population EDs provide care to children and adults in separate locations within a facility. ******* ED = emergency department. ******** This type also includes trauma centres.
